# Mathematical Identification of Critical Reactions in the Interlocked Feedback Model

**DOI:** 10.1371/journal.pone.0001103

**Published:** 2007-10-31

**Authors:** Hiroyuki Kurata, Takayuki Tanaka, Fumitaka Ohnishi

**Affiliations:** Department of Bioscience and Bioinformatics, Kyushu Institute of Technology, Fukuoka, Japan; University of Nottingham, United Kingdom

## Abstract

Dynamic simulations are necessary for understanding the mechanism of how biochemical networks generate robust properties to environmental stresses or genetic changes. Sensitivity analysis allows the linking of robustness to network structure. However, it yields only local properties regarding a particular choice of plausible parameter values, because it is hard to know the exact parameter values *in vivo*. Global and firm results are needed that do not depend on particular parameter values. We propose mathematical analysis for robustness (MAR) that consists of the novel evolutionary search that explores all possible solution vectors of kinetic parameters satisfying the target dynamics and robustness analysis. New criteria, parameter spectrum width and the variability of solution vectors for parameters, are introduced to determine whether the search is exhaustive. In robustness analysis, in addition to single parameter sensitivity analysis, robustness to multiple parameter perturbation is defined. Combining the sensitivity analysis and the robustness analysis to multiple parameter perturbation enables identifying critical reactions. Use of MAR clearly identified the critical reactions responsible for determining the circadian cycle in the *Drosophila* interlocked circadian clock model. In highly robust models, while the parameter vectors are greatly varied, the critical reactions with a high sensitivity are uniquely determined. Interestingly, not only the *per-tim* loop but also the *dclk-cyc* loop strongly affect the period of PER, although the *dclk-cyc* loop hardly changes its amplitude and it is not potentially influential. In conclusion, MAR is a powerful method to explore wide parameter space without human-biases and to link a robust property to network architectures without knowing the exact parameter values. MAR identifies the reactions critically responsible for determining the period and amplitude in the interlocked feedback model and suggests that the circadian clock intensively evolves or designs the kinetic parameters so that it creates a highly robust cycle.

## Introduction

A goal of systems biology is the generation of a coherent understanding of the mechanisms of how the integration of various components generates functional and robust systems in living organisms [1]–[3]. The importance for robustness is a functional criterion for performance of biochemical networks. Robustness is the ability to resume successful operation in the presence of signal and system uncertainties. Descriptions of the mechanisms by which biochemical networks are robust in the face of parameter uncertainty, environmental changes, and stochastic fluctuations have been published [3]–[6]. Different types of feedback loops and pathway redundancies are involved in enhanced robustness. Sensitivity analysis of mathematical models describing complex networks could allow for linking robustness properties to network structure by measuring the degree to which parametric perturbations change various target dynamics [7], [8].

In principle, both molecular architecture and the values of kinetic parameters determine the robustness of dynamic systems. In biological systems, molecular structures are being built, but it is still hard to know the exact values of kinetic parameters *in vivo*. The values of kinetic parameters vary with time and environment and the measured values *in vitro* are often different from those *in vivo*. In most studies, a particular set of local kinetic parameters has been determined for convenience so that dynamic models reproduce target data. It is necessary to exclude the possibility that the calculated results are dependent on variations in the values assigned to kinetic parameters. The dependency of robustness on parameter values has been investigated by only a few reports. To compare some performances of alternative mathematical models, Alves *et al* [9] statistically searched their parameter values so that they make the other dynamic properties the same. Stelling *et al* [10] studied dynamic properties linked to network structure in the *per-tim* feedback loop model by systematically investigating the 2D-parameter space and suggested some influential process determining the oscillator features. These previously presented random or systematic searches are a great step for approaching to global analysis, but they restricted the search space of parameters or the size of models due to calculation complexity and few criteria have been presented to determine whether the search is exhaustive.

The molecular mechanisms of how circadian clocks generate robust cycles have extensively been studied and negative feedback loops are found common structures for producing robust cycles [11]–[13]. An interlocked feedback model is typical architecture to provide robustness of the cycle and synclonization of two clocks to environmental perturbation or parameter uncertainty[14], while critical reactions responsible for determining cycle features such as amplitude and period remain to be seen due to experimental complexity. Generally both network architecture and its associated parameter values determine the system's function. For example, engineering design requires the tuning process of many parameters for producing an optimal and highly robust system. However, neither the optimal values of parameters nor the reactions critically responsible for a robust cycle are known in circadian clock systems.

Here, we propose a new strategy, named mathematical analysis for robustness (MAR), that combines robustness analysis with an evolutionary search that explores many plausible solutions for kinetic parameters producing the same dynamic properties. To search large parameter spaces without any human bias, the two-step evolutionary method is proposed and novel and rigorous criteria are introduced that characterize whether the search approaches an exhaustive level. MAR leads to an understanding of the global mechanism by which the *Drosophila* interlocked feedback system generates the robustness of the circadian cycle without insisting on the exact values of kinetic parameters.

## Results

### Mathematical Analysis for Robustness algorithm

**Figure 1:**
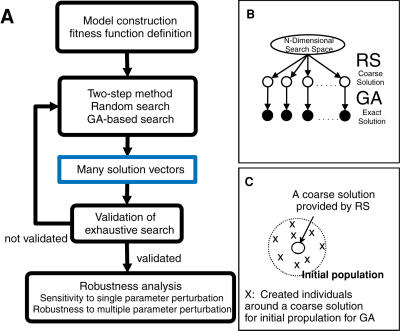
Schematic diagrams of exhaustive search and robustness analysis in MAR. A: A flowchart for MAR. B: The proposed two-step search method that consists of a random search (RS) and a search by genetic algorithms (GAs). C: How to make the initial populations for a search by GAs.

To link molecular architecture to robustness we propose MAR as shown in **Figure 1**. It consists of two stages: exhaustive search and robustness analysis. In the former stage, (1) we determine the target dynamic features that a mathematical model should simulate, (2) explore all possible solution vectors of kinetic parameters that reproduce the target features using the two-step evolutionary search, and (3) test the validity of exhaustive search (**Equations 3**, **7**). In the latter stage, (4) we simulate the sensitivity of target features to perturbation of a single parameter (**Equation 8**), (5) calculate the robustness to perturbation of multiple parameters (**Equation 13**), and (6) identify the critical reactions responsible for determining target features by combining both the sensitivity and robustness analyses. Details of the algorithms are described in Materials and Methods.

### Validation of an exhaustive search method by using a theoretical model

We propose the two-step evolutionary method that consists of the random search and the subsequent evolutionary search as a fast and non-biased method (**Figure 1BC**). To demonstrate the advantage of the two-step method, we compared it with various evolutionary searches, such as Unimordal Normal Distribution Crossover (UNDX), UNDXm, Blend Crossover (BLX), and Simplex Crossover (SPX) [15]–[17], by using a theoretical model (**Text S1** and **Equations S1-S5** in **Text S1**). The two-step method was a best choice for a fast and non-biased search among available search method (**Table S1**). To test whether the search by the two-step method becomes saturated or covers the entire solution space, we investigated the convergence of the parameters spectrum width and the variability of solutions (*VarS*) (**Equations 3**, **7**, **Figure S1**). Finally the sensitivity distributions were numerically calculated. The range of the simulated sensitivity was consistent with the theoretical range (**Equation S3** in **Text S1**) and the two-step method identified the potentially influential parameter (**Equation 10**, **Figure S2**). By using a theoretical model, the two-step method is demonstrated to explore the entire solution space at a fast speed. The parameter spectrum width and the variability of solutions are useful measures for the search process of MAR.

### Mathematical model of a biochemical oscillator and exhaustive search

**Figure 2:**
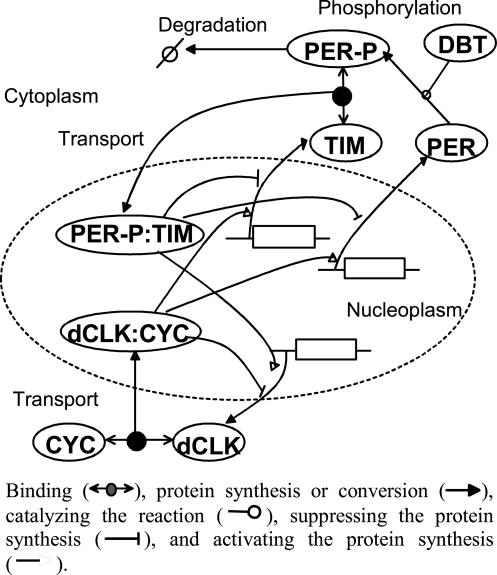
A schematic diagram of the interlocked feedback system in the *Drosophila* circadian clock. In the real biochemical reactions, the binding of PER-P:TIM to dCLK:CYC suppresses the transcription for PER and TIM and activates the transcription for dCLK. In this map, PER-P:TIM directly suppresses the transcription of PER and TIM and activates the transcription for dCLK. “:” and “-” indicate binding complexes and modification, respectively.

MAR is applied to the *Drosophila* circadian clock model as shown in **Figure 2**. We have derived mathematical equations from the interlocked-feedback model developed by Ueda et al. [14], as shown in **Tables 1** **–** [Table pone-0001103-t002] **3**. The target feature of the circadian oscillator was set as the PER oscillation curves characterized by the period and the top and bottom of the curves (amplitude). There are few reliable experimental data that measure the absolute concentrations for the circadian proteins in *Drosophila*, while they have been provided by most literatures as the relative value to a constant level molecule. Thus, we produce a mathematical but qualitative model that reproduces the intrinsic features of the circadian cycle and adjust it so that the simulated time courses of the proteins are consistent with those presented by commonly employed simulation models, e.g., the PER concentration varies from a few nM to a few dozen nM [18]. To explore the target PER oscillator, a fitness function was designed and twelve kinetic parameters were selected as the search parameters (*S[1]*, *A[1] = A[2]*, *R[1] = R[2]*,*V[1]*, *V[2]*, *D[1]*, *P[1]*, *T[1]*, *K[1]*, and *K[2]*). The two-step method was employed. Details of the fitness function and searches are described in Method (**Equations 14**, **15**) and the validity of these search parameters will be verified in the later section (Numerical sensitivity to single parameter perturbation). One thousand optimized PER oscillators were obtained by performing 2×10^5^ random searches and the subsequent GAs. We investigated the dynamic features of PER, dCLK and TIM, where the circadian model was optimized with regard to the PER cycle (**Figures S3**, **S4**). The period for dCLK or TIM was consistent with that for PER. The amplitude and mean concentration of PER oscillators varied over a narrow range of values and the ratio of the amplitude to the mean concentration was approximately 1.8. The amplitude and mean concentration of dCLK and TIM varied over a wider range of values than those for PER, and dCLK and TIM oscillated with ratios of 1.8 and 1.4, respectively. These results indicate that they are good oscillators and reproduce the target features. According to experimental data regarding the circadian oscillator, the ratio of the amplitude to the mean concentration varies from 1.2 to 2, depending on experimental conditions. These values of the ration are reasonable within the range of experimental data.

**Table 1:** Mathematical equations for the interlocked-feedback system in Drosophila

Mathematical Equations	No
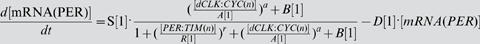	M1
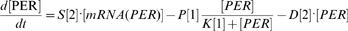	M2
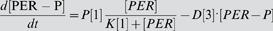	M3
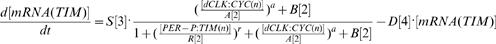	M4
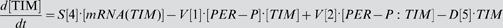	M5
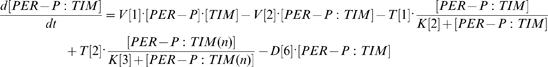	M6
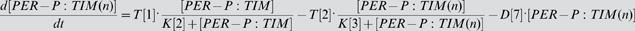	M7
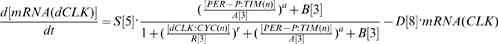	M8
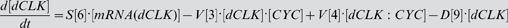	M9
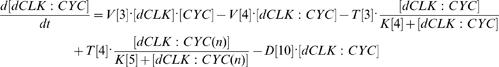	M10
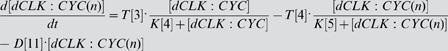	M11

**Table 2 pone-0001103-t002:** Components used in the mathematical model

Component	Definition	Concentration
*mRNA(PER)*	mRNA for PER	1.0 [nM]
*PER*	PER	1.0 [nM]
*mRNA(TIM)*	mRNA for TIM	1.0 [nM]
*TIM*	TIM	1.0 [nM]
*mRNA(dCLK)*	mRNA(dCLK)	1.0 [nM]
*dCLK*	dCLK	1.0 [nM]
*PER-P*	Phosphorylated PER	1.0 [nM]
*PER-P:TIM*	Binding complex of PER-P and TIM	1.0 [nM]
*PER-P:TIM(n)*	Binding complex of PER-P and TIM in nucleus	1.0 [nM]
*dCLK:CYC*	Binding complex of dCLK and CYC	1.0 [nM]
*dCLK:CYC(n)*	Binding complex of dCLK and CYC in nucleus	1.0 [nM]
*CYC* (constant)	CYC	1.0 [nM]

The concentration shows the initial value and n indicates that molecules are located in nucleus. “:” represents a binding complex of more than one protein; “-” represents covalent modification of a protein. Since the concentration of CYC is fixed at a constant value, there is no differential equation for CYC in **Table 1**.

**Table 3:** Kinetic parameters used in the mathematical model

Para-meter	Definition	Value	Class
*S[1]*	Transcription rate constant of PER	0.98 [nM h^−1^]	S
*S[2]*	Translation rate constant of mRNA(PER)	0.98 [h^−1^]	A
*S[3]*	Transcription rate constant of TIM	45 [nM h^−1^]	A
*S[4]*	Translation rate constant of mRNA(TIM)	0.98 [h^−1^]	A
*S[5]*	Transcription rate constant of dCLK	6.0 [nM h^−1^]	A
*S[6]*	Translation rate constant of mRNA(dCLK)	23 [M^−1^]	A
*A[1]*	DNA dissociation constant for *dCLK:CYC(n)*	0.14 [M]	S
*A[2]*	DNA dissociation constant for *dCLK:CYC(n)*	0.14 [M]	S
*A[3]*	DNA dissociation constant for *PER-P:TIM(n)*	0.21 [M]	A
*R[1]*	DNA dissociation constant for *PER-P:TIM(n)*	0.10 [M]	S
*R[2]*	DNA dissociation constant for *PER-P:TIM(n)*	0.10 [M]	S
*R[3]*	DNA dissociation constant for *dCLK:CYC(n)*	0.18 [M]	A
*B[1]*	Constant	0.71	A
*B[2]*	Constant	0.71	A
*B[3]*	Constant	0.71	A
*V[1]*	Association rate constant between PER-P and TIM	8.6 [nM^−1^ h^−1^]	S
*V[2]*	Dissociation rate constant between PER-P:TIM	0.28 [h^−1^]	S
*V[3]*	Association rate constant between dCLK and CYC	0.095 [nM^−1^ h^−1^]	A
*V[4]*	Dissociation rate constant between dCLK:CYC	0.0038 [h^−1^]	A
*T[1]*	Maximum rate constant for transportation of PER-P:TIM(cyt->nuc)	0.53 [nM h^−1^]	S
*T[2]*	Maximum rate for transportation of PER-P:TIM(nuc->cyt)	0.0 [nM h^−1^]	A
*T[3]*	Maximum rate for transportation of dCLK:CYC(cyt->nuc)	0.42 [nM h^−1^]	A
*T[4]*	Maximum rate for transportation of dCLK:CYC(nuc->cyt)	0.0 [nM h^−^1]	A
*K[1]*	Michaelis constant for phosphorylation of PER	42 [nM]	S
*K[2]*	Michaelis constant for transportation of PER-P:TIM	6.8 [nM]	S
*K[3]*	Michaelis constant for transportation of PER-P:TIM(nuc)	0.0 [nM]	A
*K[4]*	Michaelis constant for transportation of dCLK:CYC	2.3 [nM]	A
*K[5]*	Michaelis constant for transportation of dCLK:CYC(nuc)	0.0 [nM]	A
*P[1]*	Maximum rate constant for phosphorylation of PER	15 [nM h^−1^]	S
*D[1]*	Degradation rate constant of mRNA(PER)	0.56 [h^−1^]	S
*D[2]*	Degradation rate constant of PER	0.12 [h^−1^]	A
*D[3]*	Degradation rate constant of PER-P	1.2 [h^−1^]	A
*D[4]*	Degradation rate constant of mRNA(TIM)	0.60 [h^−1^]	A
*D[5]*	Degradation rate constant of TIM	0.12 [h^−1^]	A
*D[6]*	Degradation rate constant of PER-P:TIM	0.12 [h^−1^]	A
*D[7]*	Degradation rate constant of PER-P:TIM(n)	0.12 [h^−1^]	A
*D[8]*	Degradation rate constant of mRNA(dCLK)	2.3 [h^−1^]	A
*D[9]*	Degradation rate constant of dCLK	0.12 [h^−1^]	A
*D[10]*	Degradation rate constant of dCLK:CYC	0.12 [h^−1^]	A
*D[11]*	Degradation rate constant of dCLK:CYC(n)	0.12 [h^−1^]	A
*a*	Hill coefficient for activation	4	A
*r*	Hill coefficient for repressor	8	A

S: the kinetic parameters estimated by evolutionary searches, A: the values estimated/provided in the model.

### Characterization of exhaustive search

**Figure 3:**
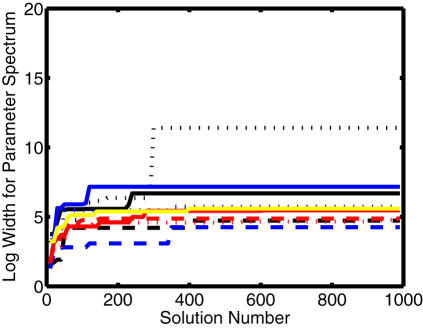
Convergence of the logarithmic width of the parameter spectrum for 12 search parameters. One thousand solutions for optimized kinetic parameters were obtained by using the repetition of the two-step search. The black solid line is *S[1]*, the black dotted line *A[1] = A[2]*, the black chain *R[1] = R[2]*, the blue solid line *V[1]*, the blue dotted line *V[2]*, the blue chain *D[1]*, the red solid line *P[1]*, the red dotted line *T[1]*, the yellow solid line *K[1]*, and the red chain *K[2]*.

To characterize whether the search is sufficient or exhaustive we investigated the convergence for two measures: the parameter-spectrum width (**Equation 3**) and the variability in the feasible solution space of the parameter vectors (**Equation 7**). **Figure 3** shows the changes in the parameter-spectrum width with respect to the number of solutions. The solutions were added by the progression of evolutionary searches. For all the search parameters, the parameter spectrum width increased with the number of solutions and then converged above a solution number of 400.

To reveal the variability of the 12-dimensional parameter vectors optimized to provide the similar time course for PER, we classified those solutions by using a hierarchical clustering with the average linkage method, where the parameter distances *D*(**P(** *i* **)**,**P(** *j* **)**) were calculated among the solutions (**Equation 4**). When the parameter spectra converged at a solution number of 400, we clustered the early 400 solutions into six super-balls, where the centroid for each cluster is employed as a representative vector.

**Figure 4:**
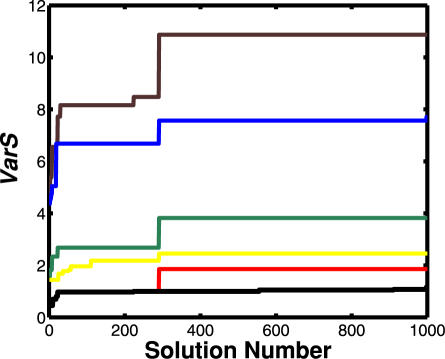
Convergence for the variability in the solutions of kinetic parameters. Six clusters were generated from using the early 400 solutions. The number of the employed clusters is changed from 1 to 6. *VarS* (Equation 7) is plotted with respect to the solution number. *VarS* of less than one shows the parameter solutions are included in the employed clusters. The brown line is one cluster employed, the blue line two clusters, the green line three clusters, the yellow four clusters, the red line five clusters, and the black line six clusters.

Here we demonstrate whether the super-balls for those clusters are sufficient for containing the subsequently optimized solutions (from 401 to 1000). As shown in **Figure 4**, we plot *VarS* (**Equation 7**) with respect to the solution number, while the number of the employed clusters is varied. This figure indicates the convergence of the variability in solution vectors with respect to the solution number. By using *VarS*, we investigate if all the solutions belong to one of the six clusters. *VarS* is less than one for the early 400 solutions when the six clusters are employed. When solutions were freshly added by the progression of evolutionary searches, the value of *VarS* increased, showing that some new solutions are distant from the centroid of the employed clusters. An increase in the number of employed clusters decreased the *VarS* value, which confirms that the increase in the employed clusters enlarges the solution space. When all the six clusters were employed, *VarS* was saturated close to one. This shows that all the solutions can be assigned to one of the employed clusters, suggesting that the search process is saturated at a solution number of 400.

### Numerical sensitivity to single parameter perturbation

**Figure 5:**
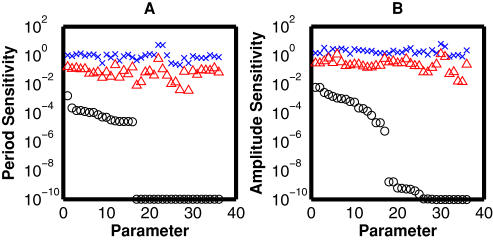
Distributions of period and amplitude sensitivities. For 400 solutions, we simulated the period and amplitude sensitivity by changing the value of each kinetic parameter by 1.5-fold. A change ratio of 1.5 was validated (data not shown). The absolute sensitivities are employed. The resulting distributions exclude the models whose oscillations are abolished by parameter perturbations (|sensitivity|>10). The sensitivities with a value of less than 10^−10^ are set to 10^−10^. The black circles indicate the minimum, the blue crosses the maximum, and the red triangles the mean, respectively. The kinetic parameters are sorted according to the minimum value of the absolute sensitivities in the descending order. Use of a threshold value of 10^−8^ separates the critically influential parameters. (A) Period sensitivity, (B) Amplitude sensitivity.

The period and amplitude sensitivities for the PER oscillator were simulated for 400 optimized solutions. The numerical sensitivity of period (*S_T_*) or amplitude (*S_A_*) to variations in a single kinetic parameter was calculated (**Equation 16**). Broad distributions of the absolute sensitivities occur as shown in **Figure 5**, where the minimum, maximum and mean values are plotted with respect to each kinetic parameter. The mean or minimum values indicate that this model provides a robust property of period and amplitude to parameter uncertainty. The period sensitivity was less than the amplitude sensitivity. Applying a threshold value of 10^−8^ to the minimum sensitivities allowed the division of kinetic parameters into potentially influential and non-influential categories (**Equation 10**). Seventeen potentially influential parameters for period and amplitude were identified respectively, which were consistent between them. PER synthesis and degradation (*S[1], S[2], D[1], D[2], D[3], D[6], D[7]*), phosphorylation and transport for PER (*P[1], K[1], T[1], K[2]*), TIM synthesis and degradation (*S[3], S[4], D[4], D[5]*), and binding of PER-P and TIM (*V[1], V[2]*) are potentially influential. It indicates that the effect of every reaction involving the *per-time* loop cannot be cancelled in any possible models. It is reasonable because the *per-tim* loop is not redundant. By contrast, the kinetic parameters involving the *dclk-cyc* loop are not potentially influential. On the other hand, a maximum value of period sensitivity was more than 0.3 (7.2 h) with respect to some kinetic parameters, indicating that not only network structures but also parameter values are critical for generating a robust cycle. If an inappropriate set of kinetic parameters is provided, an oscillation can be damaged by even a small change in a single parameter. The interlocked feedback is able to provide robustness to the circadian rhythm when the kinetic parameters are appropriately tuned.

Numerical sensitivity analysis selected 17 potentially influential parameters within the provided 12-dimensional space. A question raises whether these parameters can be potentially influential in the entire 36 dimensional parameter space. Of course, such a complete search is impossible due to calculation explosion inherent of numerical analysis. Instead, an extended search is performed to investigate how an expansion of a search parameter space affects the choice of potentially influential parameters, where the number of search parameters is varied as 3, 6, 9, 12, 16, 25, and 36. (**Table S2** and **Figure S5**). The exploration with a search parameter number of ≤9, a low-dimensional subspace, could not obtain the consistent sets of potentially influential parameters. On the other hand, the analyses with a search parameter number of ≥16 selected the same parameter sets as the 12-dimensional search. Although the variability in the solutions does not necessarily converge less than one above a search parameter number of ≥16 due to an extremely large search space, this extended search suggests that the potentially influential parameters are consistent. In conclusion, twelve search parameters are necessary and sufficient for finding them.

### Robustness to multiple parameter perturbation

There are many possible models that generate circadian oscillators. It raises a question of which models are close to a real biochemical oscillator or a question of which reactions feature robust oscillators. Robustness to multiple parameter perturbation is a key criterion for model selection. Here we define the CVs of the period and amplitude distributions generated by randomly-perturbed parameters *CV*_*Period*(*i*) and *CV*_*Amplitude*(*i*) (**Equation 13**), which characterize the robustness of the period and amplitude to simultaneous changes in all kinetic parameters, assuming that the oscillators are always exposed to uncertainty or changes in parameters under strict environmental stresses. An oscillator with a small value of CV is a highly robust model, because the change in period or amplitude is suppressed to be small against random changes in all kinetic parameters.

**Figure 6:**
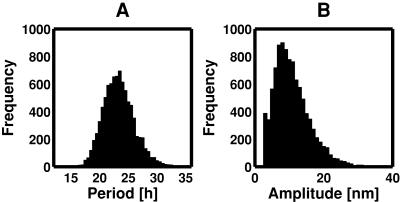
Frequency distributions of the period and amplitude in a randomly perturbed oscillator model. The period (A) and amplitude (B) were simulated 10,000 times while all kinetic parameters were randomly varied within a range from 1/1.5-fold to 1.5-fold. The oscillators with a long period (>36 h) are excluded, resulting in the remove of the oscillators with small amplitude.

**Figure 7:**
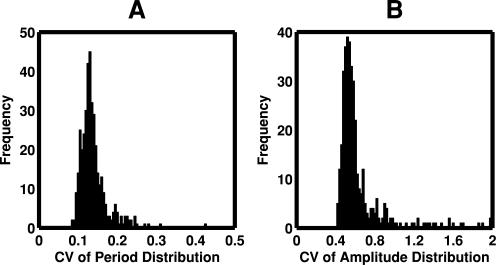
Frequency of the CVs of period and amplitude distributions in randomly perturbed oscillator models. For 400 solution vectors (oscillator models), the period and amplitude were simulated 10,000 times by randomly varying all kinetic parameters within a range from 1/1.5-fold to 1.5-fold, resulting in 400 distributions of the simulated period and amplitude. The CVs were calculated for every period and amplitude distribution and the frequency distributions of the CVs for period (A) and amplitude (B) are plotted for 400 oscillator models.

For all 400 solution vectors (oscillator models), the period and amplitude were simulated 10,000 times by randomly varying all kinetic parameters within a range from 1/1.5-fold to 1.5-fold, resulting in 400 distributions of the simulated period and amplitude. The means and CVs almost converged at 10,000 time simulations. The frequency distributions of the period and amplitude for a certain parameter vector are exemplified as shown in **Figure 6**. We plotted the frequency distribution of the CVs of period and amplitude for 400 solution vectors as shown in **Figure 7**. The CVs of period changed from 0.08 to 0.44. The mean periods were around 24 h (data not shown). A CV of 0.08 indicates 1.9 h, which significantly changes the circadian cycle. On the other hand, the CVs of amplitude changed above 0.4, which was larger than the CVs of period. The mean amplitudes were around 10 nM, which is reasonable because it is within the range of commonly simulated values [18]. This figure indicates that an appropriate selection of kinetic parameters decreases the CVs, i.e., a tuning of kinetic parameters enhances robustness to random changes in all kinetic parameters. It is interesting if a biological system adjusts kinetic parameter values so that robustness is most enhanced.

To investigate the mechanism of how the circadian clock models provide a robust property to the period and amplitude, the relationship between the CVs of period and amplitude was plotted (**Figure S6**). Significant linear correlation was not observed between the CVs of period and amplitude. The mechanism that provides a robust property to the period is suggested to be different from that to the amplitude.

### Critical parameters in highly robust oscillators

**Figure 8:**
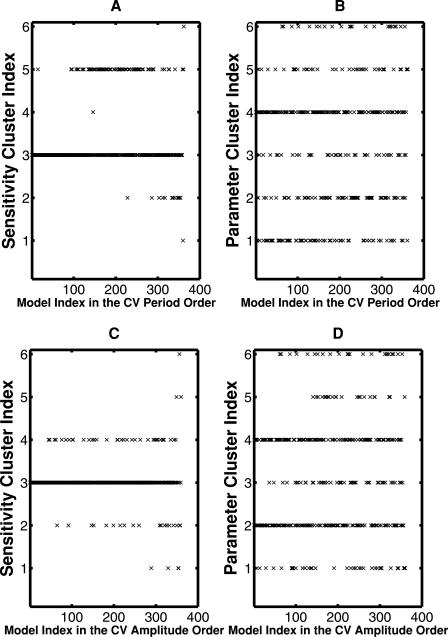
Classification of the sensitivity distribution and the parameter solution vectors. The sensitivity distributions and their associated parameter solution vectors for all the oscillator models that reproduce the circadian cycle are classified into six clusters. The six clusters for the sensitivity distributions and their associated parameter vectors are named sensitivity cluster from 1 to 6 and parameter cluster from 1 to 6, respectively. The models are sorted in the ascending order of the CV value, where the left is highly robust models because their CVs are small. The sensitivity and parameter cluster indexes that each model belongs to are plotted with respect to the model index. The sensitivity cluster (A) and parameter cluster (B) are for the models sorted according to the CV period value. The sensitivity cluster (C) and parameter cluster (D) are for the models sorted according to the CV amplitude value. Note that the number of models is less than 400 because some sensitivity distributions with a very large value (|sensitivity|>10) are excluded.

MAR combines the numerical sensitivity analysis with the CV analysis to identify the critical reactions in the interlocked feedback model. To explore the parameters that show a high sensitivity of period or amplitude, we investigated how the robustness of the oscillator models, *CV* _*Period*(*i*) or *CV*_*Amplitude*(*i*) or (**Equation 13**), is correlated to the sensitivity distributions or to the kinetic parameter vectors. The period and amplitude sensitivity distributions were classified into 6 clusters by using hierarchical clustering with the average linkage (Matlab), respectively. They were named sensitivity clusters of 1 to 6. In the same way the parameter solution vectors were classified into 6 clusters, named parameter clusters of 1 to 6. As shown in **Figure 8**, the cluster index that each model belongs to is plotted with respect to the model sorted in the ascending order of the CVs of period or amplitude.

For a high CV period (CV period order >300) the period sensitivity distributions were varied because they belonged to all the clusters. By contrast, in highly robust models with a small CV value (CV period order <50) their sensitivity distributions were consistent because most of the models belonged to the one cluster, while the parameter vectors were greatly varied (they belonged to six clusters). For a high CV amplitude (CV amplitude order >300) the amplitude sensitivity distributions were varied. In highly robust models with a small CV value (CV amplitude order <30), their sensitivity distributions were consistent because most of the models belonged to the one cluster, while the parameter vectors were different. The period and amplitude sensitivity distributions and their associated parameter vectors are illustrated (**Figures S7** and **S8**). These demonstrate that the period or amplitude sensitivity distributions are very consistent in highly robust models, while the parameter vectors are greatly varied. In other words the critical reactions are uniquely determined in highly robust models despite the variability of the kinetic parameters.

**Figure 9:**
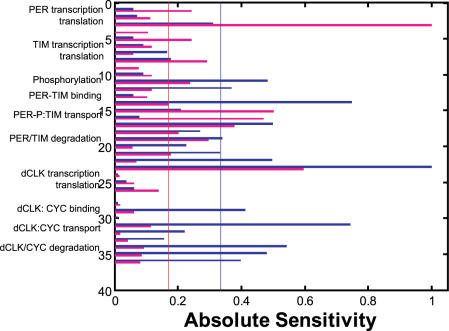
A distribution of the mean absolute sensitivity for period and amplitude in highly robust models. The blue and red bars are the period and amplitude sensitivities, respectively. The blue line is the threshold for selecting the critical parameters responsible for period and the red for amplitude. The absolute sensitivity values for ten highly robust models that show the smaller CV values are averaged. The resulting mean sensitivity is normalized by the maximum value. 1:*S[1]*, 2:*A[1]*, 3:*R[1]*, 4:*B[1]*, 5:*S[2]*, 6:*S[3]*, 7:*A[2]*, 8:*R[2]*, 9:*B[2]*, 10:*S[4]*, 11:*P[1]*, 12:*K[1]*, 13:*V[1]*, 14:*V[2]*, 15:*T[1]*, 16:*K[2]*, 17:*D[1]*, 18:*D[2]*, 19:*D[4]*, 20:*D[5]*, 21:*D[3]*, 22*:D[6]*, 23:*D[73]*, 24:*S[5]*, 25:*A[3]*, 26:*R[3]*, 27:*B[3]*, 28:*S[6]*, 29:*V[3]*, 30:*V[4]*, 31:*T[3]*, 32:*K[4]*, 33:*D[8]*, 34:*D[9]*, 35:*D[10]*, 36:*D[11]*.

Next, the critical parameters were selected from the sensitivity distributions of both period and amplitude. For ten highly robust models, we calculated the means of period and amplitude absolute sensitivities and normalized them by their maximum values, as shown in **Figure 9**. Twelve critical parameters showing a high sensitivity were selected that determine period and amplitude, respectively. The critical parameters responsible for period are *D[7], V[2], T[3], D[9], D[6], P[1], D[1], D[10], V[3], D[11], K[1], D[4];* those responsible for amplitude are *R[1], D[7], T[1], K[2], D[1], D[4], R[2], P[1], V[2], S[1], S[2], D[2].* (The parameters are sorted in the rank order of the absolute sensitivity value.) They feature highly robust oscillators (**Figures S9**).

Degradation (*D[1],D[2],D[6],D[7],D[9],D[10],D[11]*) is the key reaction that determines both the period and amplitude of the PER oscillator. Since the CVs of period were hardly correlated with those of amplitude (**Figure S6**) and the period sensitivity distributions were different from the amplitude ones, the mechanisms providing robust properties to period and amplitude are different. This is supported elsewhere [10]. Here, the kinetic parameters are classified according to their biochemical processes. The amplitude of PER can be determined mainly by reactions in the *per-tim* loop (*S[1], S[2], R[1], R[2], T[1], K[2], D[1], K[1], D[4]*), especially the synthesis and transport of PER and TIM (*S[1], S[2], R[1], R[2],T[1], K[2]*). Both *T[1]* and *K[2]* are critical, indicating transport cannot be represented as a single linear reaction. It suggests that a nonlinear reaction plays a significant role in determining the amplitude. On the other hand, the period of PER is strongly affected not only by the *per-tim* loop but also by the *dclk-cyc* loop (*D[9],D[10],D[11],V[3],T[3]*), although the *dclk-cyc* loop is neither potentially influential nor does affect the amplitude of PER. It shows that the *dclk-cyc* loop plays a critical role in determining the PER period. The phosphorylation reaction (*P[1]*, *K[1]*) would play a major role in determining the period rather than the amplitude. Both *P[1]* and *K[1]* are critical, which suggests that a nonlinear reaction is necessary for determining the period.

## Discussion

The objective of MAR is to determine critical reactions without insisting on the exact values of kinetic parameters, thereby linking robust properties to specific molecular architectures. MAR seeks global and firm results that do not depend on particular parameter values. It is a powerful tool because it is still very hard to measure full kinetics *in vivo*. The key technique is to combine robustness analysis with a two-step evolutionary search that explores many solutions for kinetic parameters showing the similar dynamic features.

Compared with the previous method that systematically analyzed two-dimensional parameter space in the *per-time* feedback model [10], MAR enables more precise analysis of more complex models, the interlocked feedback model that contains the *per-tim* and *dclk-cyc* loops. MAR is advantageous because it enables one to explore a higher dimensional and much larger space of the kinetic parameters of a complex model. Searching a larger parameter space is demonstrated to make clearer separation of potentially influential parameters (**Table S2** and **Figure S5**). Notice that a low dimensional search may find a false set of potentially influential parameters. In the circadian model, a search parameter number of ≥12 should be used to obtain the true set of potentially influential reactions in a large parameter space. This shows the extensive search is really required to obtain global and firm results from mathematical analysis.

The potentially influential parameters, which become consistent with progression of search, means that the effect of them cannot be reduced very small or less than a certain threshold value in any possible models, but they do not necessarily indicate critical parameters responsible for target instances. The potentially influential parameters would be effective in testing whether the search is exhaustive rather than in exploring critical reactions.

The central contribution here is that MAR provides the results compatible with biological knowledge or lead to new hypotheses on the function of cellular networks with realistic complexity. By searching wide parameter space, MAR gives global results that a local simulation cannot obtain. The identification of the relationship among the robustness to multiple parameter perturbation (**Equation 13**), the sensitivity distribution, and the parameter solution vectors could provide a basis to assume a potential mechanisms generating robustness. The robustness to multiple parameter perturbation greatly depends on the values of kinetic parameters. Actually, appropriate sets of kinetic parameters greatly enhance the robustness of period or amplitude, while their sensitivity values converge to a unique distribution. In highly robust models the critical reactions are uniquely determined despite the variability of the parameter values.

While the phosphorylation of PER and the transport and synthesis of PER and TIM are critical for period and for amplitude, respectively, degradations are key reactions for both period and amplitude. This outcome agrees with previous simulation studies suggesting that circadian performance is greatly affected by changes in degradation [8], [10]. Interestingly, the *dclk-cyc* loop plays a major role in determining the PER period, although the *dclk-cyc* loop is neither potentially influential nor does affect the amplitude of PER. The mechanism generating robustness to period is found different from that to amplitude. These are supported by a general theory that the robustness of one function does not imply that of other functions due to robustness tradeoff[5], [6]. Biochemical oscillators should be evolved to provide a robust property to different dynamic functions such as period and amplitude.

Engineering design conventionally consists of the investigation of functional specification and the subsequent basic and detail design. Specification is the act of understanding of a product to be modelled or of defining what a function has to achieve rather than how it has to do it. Basic design carries out the identification, classification and selection of constraints and then determines the design architecture of how the system obtains its specification. The process of detail design manipulates the system's parameters so as to satisfy the specification. In analogy to engineering design, evolution of a circadian clock is understandable in *Drosohpila*. The functional specification can be to provide robustness of the circadian cycle to various perturbations. In basic design under the constraint that the system must use genetic circuits within a cell, an interlocked feedback system is created as network architecture so that two clocks are consistently harmonized with environmental changes such as light pulse and they show robustness of the circadian period against various perturbations[14]. Generally such robustness is provided by molecular architecture such as feedback [3], [5], [6]. Then the process of detail design further evolves kinetic parameters so as to optimize the specification in the given network structure. In terms of engineering design concept, the circadian clock is suggested to not only evolve an interlocked feedback system but also intensively design kinetic parameters for further enhanced robustness.

## Materials and Methods

### General dynamic model

Dynamic models for biochemical networks are formulated as ordinary differential equations:
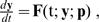
(1)where *t* is the time, **y** is the vector whose elements are the differential variables for molecular concentrations, and **p** is the vector of kinetic parameters.

### Evolutionary search for all possible solutions

The target features are determined that the dynamic model should reproduce. A fitness function must be designed so that the mathematical model reproduces the target dynamics and then all possible solutions are explored to satisfy the fitness function.

Excessive computational requirements make it impossible to explore the entire space of kinetic parameters in large-scale dynamic models. Techniques that address this problem should provide ways to reduce the number of search parameters combined with the method that allows searches within a large parameter space to proceed efficiently. In general, an evolutionary search seeks out a global minimum of certain fitness functions based on the heuristic assumption that best solutions will be found in the regions of the parameter space by using the genetic operations of selection, crossover, and mutation. However, the objective of MAR is not to find a global minimum, but to explore all possible solutions of kinetic parameter vectors that produce the target dynamics.

MAR presents the two-step evolutionary search that combines a random search with a search by GAs [17], as shown in **Figure 1**. First, the random search explores a large parameter space without any human biases and finds multiple coarse solutions showing a good fitness value. It is not necessary to find any solutions providing highest fitness values. The coarse solutions are employed to make initial populations for the subsequent GAs. Second, each initial population for GAs is created around one of the coarse solution vectors. The search by GAs focuses on the space surrounding the coarse solution and is applied to each initial population independently to find plausible solution vectors that show a high fitness value, i.e., provide the target features. The search is performed by various crossover methods, resulting in obtaining local solutions around the coarse solutions. The *i*-th resultant solution vector of kinetic parameters **P(** *i* **)** is given by:
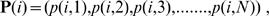
(2)where *p(i, k)* is the value of the *k*-th parameter of the *i*-th solution vector and *N* is the number of search parameters.

### Characterization of exhaustive search

The proposed two-step method explores multiple plausible solution vectors for kinetic parameters that generate target features or satisfy a fitness function. It is necessary to characterize whether the search is saturated or exhaustive. Here saturation means that the variability in all possible solutions is large enough to cover almost the entire solution space. Since numerical methods are not theoretically able to guarantee the exhaustive search, we present practically useful criteria: a parameter spectrum width and the variability in the solution vectors for parameters.

First, a parameter spectrum is defined as the allowable range of each parameter. The range is from the minimum value to the maximum for the resultant parameter solutions. The logarithmic width of the parameter spectrum for each kinetic parameter is defined by:
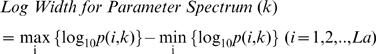
(3)where *La* is the number of all the solutions. The parameter spectrum width can be regarded as the indicator for a large-space search. Convergence of the width with respect to the number of generated solution vectors suggests that the search is approaching saturation. The number of the solutions at which the spectrum width begins to saturate is defined as *Ls*.

Second, to reveal the variability in the solution vectors that show the target dynamic behaviours, the distance between two *N-*dimensional solution vectors (**P(** *i* **)**,**P(** *j* **)**) is defined by:
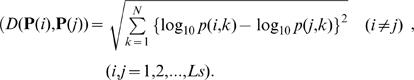
(4)We classify the solution vectors from *1* to *Ls* by using hierarchical clustering with the average linkage. The number of clusters (*M*) is determined by inspecting the dendrogram. Each cluster is named *C*(*j*), (*j* = *1,2,…M*). Assuming that the clusters form *N*-dimensional super-balls, we define the radius of each cluster as follows:
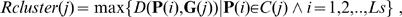
(5)where **G(** *j* **)** = (*g(j,1),g(j,2),g(j,3),…g(j,N)*) is the centroid for *C*(*j*) and *g*(*j, k*) is the *k*-th parameter of the centroid vector. The distances between the centroid and intra-cluster parameter vectors are calculated and the maximum distance is set to the radius of the cluster ball.

Third, to investigate whether the solution vectors, {**P(** *i* **)**|*i = 1,2,…,La*}, can be assigned to one of the existing cluster balls, we define the relative minimum distance between **P(** *i* **)** and the centroid of each cluster as follows:
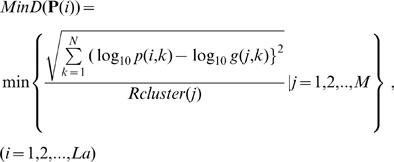
(6)where the relative distance between **P(** *i* **)** and each **G(** *j* **)** is calculated and the relative minimum distance is selected as *MinD*(**P**(*i*)). *MinD*(**P**(*i*)) determines whether a solution vector **P(** *i* **)** belongs to one of the existing cluster balls or not. When *MinD*(**P**(*i*)) is less than one, **P(** *i* **)** belongs to one of the existing clusters. When *MinD*(**P**(*i*)) is greater than one, it does not any cluster.

Finally, to display the convergence of the variability in the solution vectors, we define *VarS(L)* as the maximum distance of {*MinD*(**P** **(** *i* **)**)|*i = 1,2,…L*}:
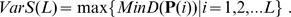
(7)
*VarS(L)* is defined as an indicator of whether the *L* parameter solutions fall into one of the cluster-balls generated by the *Ls* solutions. When *VarS(L)* is less than one, the *L* solution vectors are assigned to one of the existing clusters, indicating that the cluster-balls contain all *L* solutions. In contrast, when *VarS(L)* is greater than one, these clusters are not able to contain all *L* solutions. Convergence of *VarS* to less than one indicates that the cluster-balls are able to cover the parameter space spanned by the *L* solution vectors. In other words, *Ls* is large enough to provide the variability in the solutions and the evolutionary search is suggested to approach saturation at a solution number of *Ls*.

### Numerical sensitivity to single parameter perturbation

It is important to note which structural characteristics of specific molecular networks are responsible for specific instances of robustness. The numerical sensitivity of a target instance to variations in a single kinetic parameter *p(i, k)* is defined as follows:
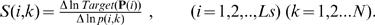
(8)Sensitivity analysis contributes to finding critical reactions for determining the target feature, in which kinetic parameters showing high sensitivities are critical.

In MAR the potentially influential parameters for determining a specific target feature are defined. The minimum sensitivity values provided by:
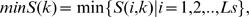
(9)are compared for all kinetic parameters and the potentially influential parameters (*C*) are selected by:
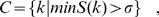
(10)where *k* is the index of the selected parameters and σ is the threshold value that is determined from the distribution of *minS*(*k*). The threshold value depends on mathematical models or target instances. The numerical sensitivity for the potentially influential parameters cannot be reduced less than a threshold value of σ, while that for the other parameters can be reduced.

### Robustness to multiple parameter perturbation

There are many possible models that produce the target instance. It raises a question of which models are close to a real biochemical model. Robustness to multiple parameter perturbation is a key criterion for model selection. The intracellular environment consistently varies with time and with changes in external stresses, which would provide uncertainty to kinetics for all intracellular molecules. To characterize the changes in a target instance with respect to simultaneous changes in all kinetic parameters, *Target*(**p** *_r_*(*i*)) are simulated by randomly varying all kinetic parameters of **p**(*i*)(*i = 1,2,…,Ls*) within a specific range given by *ρ*, where **p** *_r_*(*i*) = (*p_r_(i,1),p_r_(i,2),…p_r_(i,N)*) (*r = 1,2,…,Nr*) is the randomly-perturbed parameter vector and *Nr*indicates the repetition number of the random simulations. The component of **p**_r_(*i*) is given by:*p_r_*(*i,k*) = γ(*r,i,k*) · *p*(*i,k*), (*k* = *1,2,…,N*) where γ(*r,i,k*) is the uniform random value within a range of:
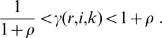
(11)By simulating *Target*(**p**_r_(*i*)), the frequency distribution of *Target*(**p**_r_(*i*)) occurs. To characterize its distribution, we define the mean and coefficient of variance (CV) by:
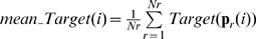
(12)

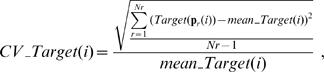
(13)respectively. A small value of CV indicates that a distribution is narrow and the model provides a robust property to simultaneous changes in all kinetic parameters.

### Link of reactions to robustness

Finally MAR combines the sensitivity distribution *S*(*i,k*) (*k = 1,2,…,N*) and *CV*_*Target*(*i*) with respect to **p**(*i*) to explore the critical reactions responsible for determining the target instance. For highly robust models with a small value of *CV*_*Target*(*i*), the parameters showing a high sensitivity are selected as critical parameters. Use of statistical methods investigates how the selected critical parameters feature highly robust models, thereby linking reactions to robustness.

### Biochemical model

Circadian rhythms are defined as endogenously generated 24-h variations in behaviour and physiology that allow organisms to adapt to the varying environmental demands of the solar cycle. In *Drosophila*, oscillating levels of period (*per*) and timeless (*tim*) constitute the fly's molecular clock. The circadian feedbacks are composed of two interlocked negative feedback loops: a *per-tim* loop, which is activated by *Drosophila* clock (*dclk*) and repressed by PER:TIM, and a *dclk-cyc* loop, which is repressed by dCLK:CYC and derepressed by PER:TIM[12]. A loop that connects *per-tim* to *dclk-cyc* represents a positive feedback loop. **Figure 2** shows a schematic diagram of the circadian clock, with the notation of the reactions described elsewhere [19]. PER and TIM form dimers and then inhibit transcription of their own genes. Expression of the *per* and *tim* genes is regulated by a pair of transcription factors, dCLK and CYC, whose activity is decreased by forming a complex with PER:TIM [20]. Phosphorylation and proteolysis of PER make a time-delayed negative feedback, generating oscillations. PER is phosphorylated by DBT (encoded by the double-time (*dbt*) gene), which is present at roughly constant levels during the rhythm [21]. Phosphorylated PER (PER-P) is readily susceptible to degradation, while PER-P is stabilized by dimerization with TIM. The dimerized PER-P:TIM is transported to the nucleus. Transcription of *dclk* and *cyc* is regulated by PER-P:TIM-mediated release of dCLK:CYC-dependent repression.

### Mathematical model

We have derived mathematical equations from the interlocked-feedback model developed by Ueda et al. [14]. In Ueda's model, while PER directly binds to TIM, it is not considered that phosphorylated PER binds to TIM. Thus, we present the modified mathematical model that includes the mechanism for DBT-phosphorylated PER binding to TIM (**Tables 1** **–** [Table pone-0001103-t002] **3**). DBT does not appear in the model as it is assumed that the DBT concentration is constant. The modified model is described by 11 ordinary differential equations. The CADLIVE system is employed for numerical simulation (http://www.cadlive.jp) [17].

### Optimization for the mathematical model

The dynamic model of the circadian system has 36 kinetic parameters to search as shown in **Table 3**, where *T[2], T[4], K[3],* and *K[5]* are set to zero to reduce the number of search parameters. Twelve parameters (*S[1], A[1], A[2], R[1], R[2], V[1], V[2], D[1], P[1], T[1], K[1], K[2]*) that are related to feedback architectures were explored using the evolutionary search in order to generate stable oscillators for PER. These parameters involve the transcription, phosphorylation, transport, and degradation of PER and the binding between PER-P and TIM. The values of the remaining parameters are estimated or provided from the previous model [14].

Based on the two-step method we randomly vary the values of 12 parameters in logarithmic space to find parameter solutions that indicate some oscillations, where a basis parameter vector is determined that produces a circadian oscillator and the value of each basis parameter is 10^−2^ to 10^2^-fold varied. Such solutions will be employed as the initial population for the subsequent search by GAs. In the random search, an oscillation curve of PER is sampled after a simulation time of 300 h and the fitness function is provided by:
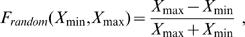
(14)where *X_min_* is the minimum value of an oscillation curve of PER and *X_max_* is its maximum. At this stage, it is important to find the coarse solutions showing some oscillations because GAs cannot evolve non-oscillatory solutions into stable oscillators. The initial population with 100 individuals is created around the coarse solution that shows some oscillatory behaviors.

Next, to search for the solutions that show stable oscillations with a 24-h period and large amplitude, GAs are carried out in logarithmic space, where each coarse solution obtained by the random search is set to the basis parameters and the value of each basis parameter is 10^−1^ to 10-fold varied. Twelve kinetic parameters are optimized where the generations and population are set to 100 and 100, respectively. The Unimordal Normal Distribution Crossover (UNDX) is employed as crossover [17], while mutation is not used. Twenty elites are selected for each generation. The cycle features provide the fitness function:
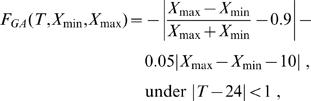
(15)where *T* is the period. **Equation 15** is an experience formula that generates the 24-h cycles whose amplitude is large enough for the average of the oscillatory curves. A target PER oscillator is that the ratio of the amplitude to the mean concentration of PER (the first term in **Equation 15** is 1.8 (0.9×2), the amplitude (the second term) is 10 nM, and the period is between 23 and 25 h.

### Robustness analysis for the biochemical model

The numerical sensitivity of period (*S_T_*) or amplitude (*S_A_*) to variations in a single kinetic parameter is calculated by:
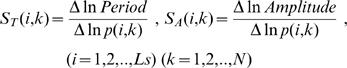
(16)where *Ls* and *N* are set to 400 and 36, respectively. Each kinetic parameter was changed by 1.5-fold. In robustness analysis to multiple parameter perturbation, characterized by the CVs of period and amplitude, the period and amplitude are simulated 10,000 times by randomly varying all kinetic parameters within a range from 1/1.5-fold to 1.5-fold (*ρ* = 0.5) for *Ls* models. The means and CVs: *mean_Period(i)*, *CV_Period(i)*, *mean_Amplituede(i)*, and *CV_Amplitude(i)*, are calculated for all the distributions(*i* = 1,2,…*Ls*).

### Computation

Since a search parameter space is very large, the obigrid system (RIKEN, Yokohama) is used to greatly increase a calculation speed, where dozens of computers are available simultaneously[22]. Dynamic simulation programs are written in C. The Runge-Kutta method is employed.

### Statistical analysis

A hierarchical clustering method with the average distance is used to classify parameter solution vectors. The Matlab functions, linkage and dendrogram, are employed to determine the number of clusters. Other statistical analyses are also carried out by Matlab.

## Supporting Information

TextS1 (0.08 MB PDF)

TableS1 (0.13 MB PDF)

TableS2 (0.11 MB PDF)

FigureS1 (0.11 MB PDF)

FigureS2 (0.09 MB PDF)

FigureS3 (0.10 MB PDF)

FigureS4 (0.12 MB PDF)

FigureS5 (0.14 MB PDF)

FigureS6 (0.11 MB PDF)

FigureS7 (0.10 MB PDF)

FigureS8 (0.10 MB PDF)

FigureS9 (0.12 MB PDF)

## References

[1] Savageau MA (1971). Concepts relating the behavior of biochemical systems to their underlying molecular properties.. Arch Biochem Biophys.

[2] Savageau MA (1971). Parameter sensitivity as a criterion for evaluating and comparing the performance of biochemical systems.. Nature.

[3] Csete ME, Doyle JC (2002). Reverse engineering of biological complexity.. Science.

[4] El-Samad H, Kurata H, Doyle JC, Gross CA, Khammash M (2005). Surviving heat shock: control strategies for robustness and performance.. Proc Natl Acad Sci U S A.

[5] Kurata H, El-Samad H, Iwasaki R, Ohtake H, Doyle JC (2006). Module-based analysis of robustness tradeoffs in the heat shock response system.. PLoS Comput Biol.

[6] Stelling J, Sauer U, Szallasi Z, Doyle FJ, Doyle J (2004). Robustness of cellular functions.. Cell.

[7] Ma L, Iglesias PA (2002). Quantifying robustness of biochemical network models.. BMC Bioinformatics.

[8] Bagheri N, Stelling J, Doyle FJ (2007). Quantitative performance metrics for robustness in circadian rhythms.. Bioinformatics.

[9] Alves R, Savageau MA (2000). Extending the method of mathematically controlled comparison to include numerical comparisons.. Bioinformatics.

[10] Stelling J, Gilles ED, Doyle FJ (2004). Robustness properties of circadian clock architectures.. Proc Natl Acad Sci U S A.

[11] Leloup JC, Goldbeter A (2000). Modeling the molecular regulatory mechanism of circadian rhythms in *Drosophila*.. Bioessays.

[12] Glossop NR, Lyons LC, Hardin PE (1999). Interlocked feedback loops within the Drosophila circadian oscillator.. Science.

[13] Cheng P, Yang Y, Liu Y (2001). Interlocked feedback loops contribute to the robustness of the *Neurospora* circadian clock.. Proc Natl Acad Sci U S A.

[14] Ueda HR, Hagiwara M, Kitano H (2001). Robust oscillations within the interlocked feedback model of *Drosophila* circadian rhythm.. J Theor Biol.

[15] Ono I, Kobayashi S (1997). A Real-coded Genetic Algorithm for Function Optimization Using Unimodal Normal Distribution Crossover.. Proc of 7th Int Conf on Genetic Algorithms.

[16] Eshelman LJ, Schaffer JD, Whitley LD (1993). Real-coded genetic algorithms and interval schemata.. Foundations of Genetic Algorithms.

[17] Kurata H, Masaki K, Sumida Y, Iwasaki R (2005). CADLIVE Dynamic Simulator: Direct Link of Biochemical Networks to Dynamic Models.. Genome Res.

[18] Smolen P, Baxter DA, Byrne JH (2001). Modeling circadian oscillations with interlocking positive and negative feedback loops.. J Neurosci.

[19] Kurata H, Matoba N, Shimizu N (2003). CADLIVE for constructing a large-scale biochemical network based on a simulation-directed notation and its application to yeast cell cycle.. Nucleic Acids Res.

[20] Stanewsky R (2003). Genetic analysis of the circadian system in *Drosophila melanogaster* and mammals.. J Neurobiol.

[21] Suri V, Hall JC, Rosbash M (2000). Two novel doubletime mutants alter circadian properties and eliminate the delay between RNA and protein in *Drosophila*.. J Neurosci.

[22] Kimura S, Kawasaki T, Hatakeyama M, Naka T, Konishi F (2004). OBIYagns: a grid-based biochemical simulator with a parameter estimator.. Bioinformatics.

