# Supplementary material for: Mathematical Identification of Critical Reactions in the Interlocked Feedback Model
Source: PLoS One. 2007 Oct 31;2(10):e1103. doi: 10.1371/journal.pone.0001103 (PMC2040204; doi:10.1371/journal.pone.0001103)
Supplement: TextS1 — (0.08 MB PDF) [file pone.0001103.s001.pdf]

Text S1 A theoretical model for validation of the exhaustive search in MAR

To demonstrate the validity of the exhaustive search of MAR, we present a theoretical model with an autogeneous negative feedback model:

$$\frac{dy}{dt} = k\frac{K}{K+y} - y, \qquad \text{(S1)}$$

where y is the protein concentration, k the synthesis rate constant and K the binding constant. The protein suppresses the synthesis of itself. The steady-state concentration y_s is analytically provided by:

$$y_s = \frac{-K+\sqrt{K^2+4kK}}{2}. \qquad \text{(S2)}$$

The logarithmic gain of the steady-state concentration with respect to a kinetic parameter (sensitivity) is analytically solved and the range of the sensitivity is provided by:

$$0 < \left.\frac{\partial \ln y}{\partial \ln K}\right|_{y=y_s} < 0.5, \quad 0.5 < \left.\frac{\partial \ln y}{\partial \ln k}\right|_{y=y_s} < 1. \qquad \text{(S3)}$$

The target behavior is set to a steady-state concentration y_s of 1. The fitness function that the two-dimensional parameters (*k, K*) must satisfy is defined by:

$$F = -\left|y_s - 1\right|. \qquad \text{(S4)}$$

The fitness value is constrained by:

$$\left|F\right| < \varepsilon \;. \qquad \text{(S5)}$$

where ε is the allowable error.

The kinetic parameters of the theoretical model of **Equation S1** are optimized by various evolutionary methods and a random search. Two kinetic parameters (*k, K*) are explored in logarithmic space, where the basis parameter vector is set to $(k,K)=(2,1)$ that provides one to the steady state concentration of y_s and the allowable error is set to 0.0001. The fitness function of **Equation S4** is employed with an allowable error of **Equation S5**. In the random search (control), the value of each basis parameter was 10^{-2} to 10^{2}-fold varied randomly in logarithmic space. In the two-step search, a random search was first performed to obtain the coarse solutions with an allowable error of 0.1. Subsequently GA grew up the coarse solutions independently. The coarse solution was set to the basis parameters and the value of each basis parameter was 10^{-1} to 10-fold varied randomly for the initial population for the GA. The population number was 10 and the generation number of 10. The Unimodal Normal Distribution Crossover (UNDX) was employed as the crossover method. Five elites were selected for each generation. The other evolutionary searches (UNDX, UNDXm, BLX, and SPX) employed the same logarithmic space as by the random search, where the population number was 10 and the generation number of 10. Mutation was not employed so that GAs intensively searched the neighborhood of the coarse solution.
