# Supplementary material for: Mathematical Identification of Critical Reactions in the Interlocked Feedback Model
Source: PLoS One. 2007 Oct 31;2(10):e1103. doi: 10.1371/journal.pone.0001103 (PMC2040204; doi:10.1371/journal.pone.0001103)
Supplement: TableS1 — (0.13 MB PDF) [file pone.0001103.s002.pdf]

Table S1 Comparison of performance of the two-step evolutionary method with other methods
Allowable error was 0.0001. The search performance was characterized by the calculation repetition number, and the mean and CV of the solution distributions. The random search was employed as a control method.

	Repetition number	mean(*k*)	mean(*K*)	CV(*k*)	CV(*K*)
**RS (control)**	**30300**	**16.9**	**4.00**	**1.35**	**2.75**
**Two-step**	**1700**	**16.9**	**4.01**	**1.35**	**2.89**
SPX	10300	12.9	4.20	1.61	2.98
UNDXm	7800	10.3	4.15	1.84	2.80
UNDX	1200	6.49	0.910	1.07	1.52
BLX	1100	7.62	7.33	1.81	2.16

For a search speed, the average repetition number of simulations that was required to find one solution was defined. The repetition number was calculated by the number of simulations being divided by the number of solutions. For the distribution of the optimized solutions, the mean and coefficient of variance (CV) of them were calculated. The mean and CV were calculated for the kinetic parameters (*k, K*) of **Equation S1**. A large value of CV reflects that the solutions disperse. The mean and CV of the solution distribution by the two-step search was most close to those by random search (RS), indicating that the proposed two-step method searches a given space without falling into local points, i.e., without little biases, as well as RS. SPX and UNDXm provided a larger repetition number than the two-step search. SPX and UNDXm were poor methods in terms of a calculation speed. The repetition numbers by UNDX and BLX were smaller than that by the two-step search, while the mean and CV of them are far different from those of RS. In terms of non-biased search, the two-step search was better than UNDX and BLX.

The repetition number of random searches is suggested to be important for non-biased search, because the total number of random searches carried out by the two-step method is larger than those of the UNDX and BLX methods. The solution distributions by such ordinary GA algorithms are biased compared with a random search with an increase in a calculation speed. A rapid optimization may increase the probability that a chromosome population falls into local minimums. In the two-step method, the extensive random search leads to non-biased search and the subsequent GA enables a rapid optimization.
