# Supplementary material for: Mathematical Identification of Critical Reactions in the Interlocked Feedback Model
Source: PLoS One. 2007 Oct 31;2(10):e1103. doi: 10.1371/journal.pone.0001103 (PMC2040204; doi:10.1371/journal.pone.0001103)
Supplement: TableS2 — (0.11 MB PDF) [file pone.0001103.s003.pdf]

Table S2 Effect of the number of search parameters on selection of potentially influential parameters
The table derives from **Figure S5**, where details in the simulation method are described. Application of a threshold value of 10^{-8} to the amplitude sensitivities separates the potentially influential parameters (**Equation 10**), as marked in boldface. The kinetic parameters are sorted according to the minimum sensitivities in the descending order. The numbers of search parameters are 3 (A), 6 (B), 9 (C), 12 (D), 16 (E), 25 (F), and 36 (F), respectively. For a search parameter number of ≥ 12, the members of the seventeen potentially influential parameters are the same.

**A**									**3**								
R[1]	***D[7]***	***A[2]***	***T[1]***	***K[2]***	***D[4]***	***R[3]***	***T[3]***	***D[10]***	***D[9]***	***D[11]***	***S[4]***	***S[3]***	***A[3]***	***D[1]***	***R[2]***	***V[2]***	***P[1]***
D[8]	***V[3]***	***D[3]***	***D[6]***	***S[5]***	***S[6]***	***D[2]***	***V[4]***	***A[1]***	***V[1]***	***S[2]***	***S[1]***	***D[5]***	***K[1]***	***K[4]***	*B[3]*	*B[2]*	*B[1]*
**B**									**6**								
K[2]	***D[7]***	***D[1]***	***T[1]***	***P[1]***	***D[2]***	***R[1]***	***D[3]***	***V[2]***	***S[2]***	***S[1]***	***S[3]***	***S[4]***	***R[2]***	***D[6]***	***D[4]***	***D[5]***	***K[1]***
V[1]	***R[3]***	***T[3]***	***D[9]***	***D[10]***	***A[2]***	***D[11]***	***A[3]***	***V[3]***	***D[8]***	***S[5]***	***S[6]***	***K[4]***	*A[1]*	*V[4]*	*B[1]*	*B[2]*	*B[3]*
**C**									**9**								
D[7]	***K[2]***	***S[4]***	***S[3]***	***R[1]***	***T[1]***	***D[3]***	***D[5]***	***P[1]***	***R[2]***	***D[6]***	***D[1]***	***D[2]***	***K[1]***	***S[1]***	***S[2]***	***D[4]***	***V[2]***
V[1]	*S[5]*	*S[6]*	*A[1]*	*A[2]*	*A[3]*	*R[3]*	*B[1]*	*B[2]*	*B[3]*	*V[3]*	*V[4]*	*D[8]*	*D[9]*	*D[10]*	*D[11]*	*T[3]*	*K[4]*
**D**									**12**								
D[1]	***T[1]***	***D[4]***	***D[3]***	***S[3]***	***S[4]***	***D[5]***	***P[1]***	***D[7]***	***S[1]***	***S[2]***	***V[1]***	***K[1]***	***D[6]***	***V[2]***	***D[2]***	***K[2]***	*R[3]*
T[3]	*D[9]*	*D[10]*	*A[2]*	*A[1]*	*D[8]*	*D[11]*	*S[6]*	*S[5]*	*A[3]*	*R[1]*	*R[2]*	*B[1]*	*B[2]*	*B[3]*	*V[3]*	*V[4]*	*K[4]*
**E**									**16**								
S[1]	***S[2]***	***D[1]***	***K[1]***	***D[4]***	***D[3]***	***P[1]***	***S[3]***	***S[4]***	***D[2]***	***D[6]***	***D[5]***	***T[1]***	***V[1]***	***D[7]***	***V[2]***	***K[2]***	*R[3]*
K[4]	*S[6]*	*A[3]*	*D[11]*	*V[3]*	*T[3]*	*A[1]*	*D[9]*	*D[10]*	*S[5]*	*A[2]*	*R[1]*	*R[2]*	*B[1]*	*B[2]*	*B[3]*	*V[4]*	*D[8]*
**F**									**25**								
D[4]	***T[1]***	***K[2]***	***P[1]***	***D[7]***	***S[3]***	***K[1]***	***D[1]***	***D[2]***	***S[2]***	***V[1]***	***D[3]***	***D[6]***	***V[2]***	***S[1]***	***D[5]***	***S[4]***	S[5]
S[6]	*A[1]*	*A[2]*	*A[3]*	*R[1]*	*R[2]*	*R[3]*	*B[1]*	*B[2]*	*B[3]*	*V[3]*	*V[4]*	*D[8]*	*D[9]*	*D[10]*	*D[11]*	*T[3]*	*K[4]*
**G**									**36**								
D[1]	***D[3]***	***K[1]***	***S[2]***	***P[1]***	***S[1]***	***D[4]***	***S[3]***	***S[4]***	***D[5]***	***V[1]***	***D[7]***	***T[1]***	***D[2]***	***K[2]***	***D[6]***	***V[2]***	*S[5]*
S[6]	*A[1]*	*A[2]*	*A[3]*	*R[1]*	*R[2]*	*R[3]*	*B[1]*	*B[2]*	*B[3]*	*V[3]*	*V[4]*	*D[8]*	*D[9]*	*D[10]*	*D[11]*	*T[3]*	*K[4]*
