# Supplementary material for: Mathematical Identification of Critical Reactions in the Interlocked Feedback Model
Source: PLoS One. 2007 Oct 31;2(10):e1103. doi: 10.1371/journal.pone.0001103 (PMC2040204; doi:10.1371/journal.pone.0001103)
Supplement: FigureS1 — (0.11 MB PDF) [file pone.0001103.s004.pdf]

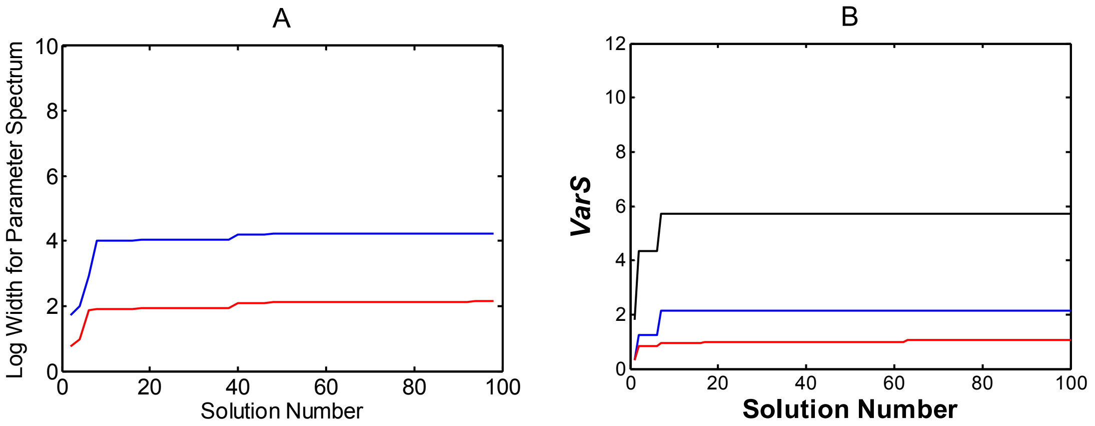

Figure S1 Convergence of parameter solutions by the two-step method in a theoretical model
(A) Convergence of the logarithmic width of the parameter spectrum for two parameters. One hundred of solutions for optimized kinetic parameters were obtained by using the repetition of the two-step search. The blue line is *K* and the red line *k*. (**Equation S1**)
(B) Convergence for the variability in the solutions of kinetic parameters. Three clusters were generated from using the early 40 solutions. The number of the employed clusters is changed from 1 to 3. *VarS* (**Equation 7**) is plotted with respect to the solution number. *VarS* of less than one shows that the generated solutions are included in the employed clusters. The black line is one cluster employed, the blue line two clusters, and the red line three clusters.

The parameter spectrum width spreads with an increase in the solution number, indicating that the variability in the solution vectors becomes large. The spectrum width is almost saturated above 40 (**Figure S1A**). A hierarchical clustering with the average linkage method is applied to the initial 40 solutions, classifying the solution vectors into three clusters. By using three clusters, we investigate the convergence for the *VarS* value as shown in **Figure S1B**. The *VarS* value decreases with an increase in the number of the employed clusters. When all the three clusters are used, the *VarS* value begins to converge close to one at 40. The two-step method approaches saturation or covers almost the entire solution space at a solution number of 40 in the given search parameter space.
