# Supplementary material for: Mathematical Identification of Critical Reactions in the Interlocked Feedback Model
Source: PLoS One. 2007 Oct 31;2(10):e1103. doi: 10.1371/journal.pone.0001103 (PMC2040204; doi:10.1371/journal.pone.0001103)
Supplement: FigureS2 — (0.09 MB PDF) [file pone.0001103.s005.pdf]

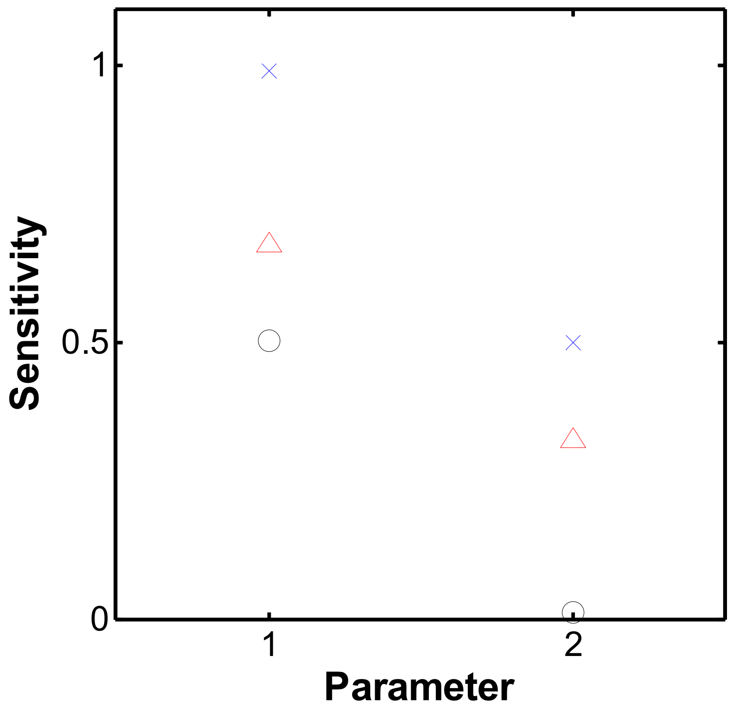

Figure S2 A sensitivity distribution of two kinetic parameters in a theoretical model
The sensitivity distributions were numerically calculated by changing each kinetic parameter by one percentage. Forty solutions are employed. The mean (red triangle), minimum (black circle), and maximum (blue cross) values are plotted with respect to each kinetic parameter. 1 is k and 2 is K.

The range of the simulated sensitivity is consistent with the theoretical range (**Equation S3**), showing that MAR predicts the sensitivity range precisely. The sensitivity for k cannot be suppressed to be less than 0.5, whereas that for K can be reduced to zero. When the threshold value is set to 0.5, the parameter of k is the potentially influential parameter responsible for the steady-state level.
