# Supplementary material for: Mathematical Identification of Critical Reactions in the Interlocked Feedback Model
Source: PLoS One. 2007 Oct 31;2(10):e1103. doi: 10.1371/journal.pone.0001103 (PMC2040204; doi:10.1371/journal.pone.0001103)
Supplement: FigureS3 — (0.10 MB PDF) [file pone.0001103.s006.pdf]

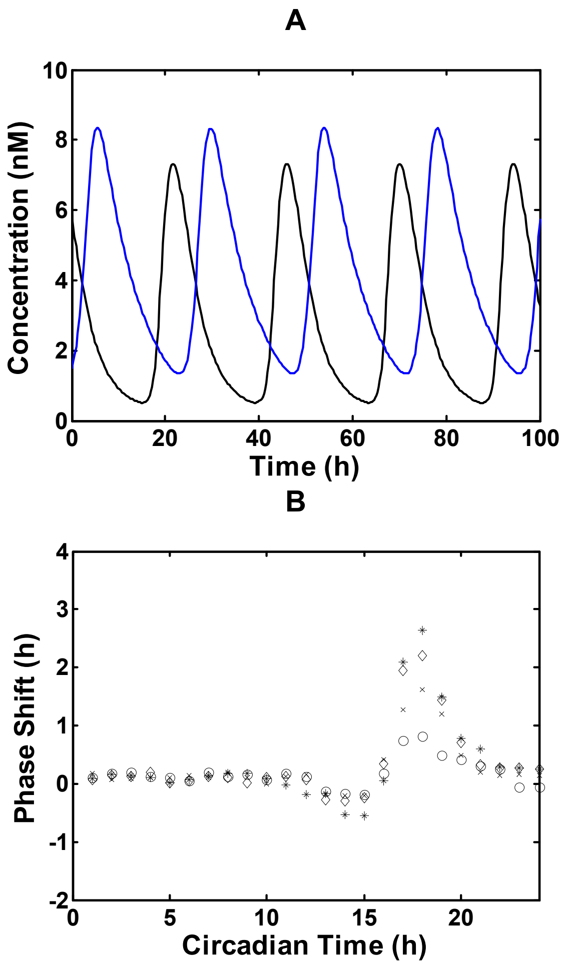

Figure S3 Dynamic features for the interlocked feedback model

(A) Time evolution for the total concentrations for PER and dCLK. The black curve is PER and the blue curve is dCLK. The model reproduces the typical experimental features that the time evolutions for PER and dCLK oscillate in turn. There are few experimental data regarding the absolute concentration of the circadian proteins. The simulated time courses of the proteins are consistent with those presented by commonly used mathematical models, e.g., the PER and dCLK concentrations vary from a few nM to a few dozen nM [1].

(B) Light pulse-induced phase shift of the peak time for mRNA for PER. Since TIM is degraded by light, we emulate light pulse by increasing the degradation rate constant (*D[5]*) for TIM by 2- (open circle), 4- (cross), 8-(diamond), or 16-fold (star) for 1 h. We apply the same promotion of TIM degradation to the model on 24 different circadian time points and measure the following peak time of *per* mRNA. By comparing it with the peak time of *per* mRNA without perturbation, we calculate the phase shifts.

The phase shifts occurs depending on the circadian time at which perturbation is applied. The simulated results are consistent with the observation that light-pulses delay the phase of the circadian activity rhythms during early subjective night and advance the phase during late subjective night whereas light pulses tend to cause minimal or no phase shifts during the subjective day [2], although the simulated phase delay at 15 h is smaller than the experimental observation.

References

1. Smolen P, Baxter DA, Byrne JH (2001) Modeling circadian oscillations with interlocking positive and negative feedback loops. J Neurosci 21: 6644-6656.
2. Hall JC, Rosbash M (1987) Genes and biological rhythms. Trends Genet 3: 185-191.
