# Supplementary material for: Mathematical Identification of Critical Reactions in the Interlocked Feedback Model
Source: PLoS One. 2007 Oct 31;2(10):e1103. doi: 10.1371/journal.pone.0001103 (PMC2040204; doi:10.1371/journal.pone.0001103)
Supplement: FigureS4 — (0.12 MB PDF) [file pone.0001103.s007.pdf]

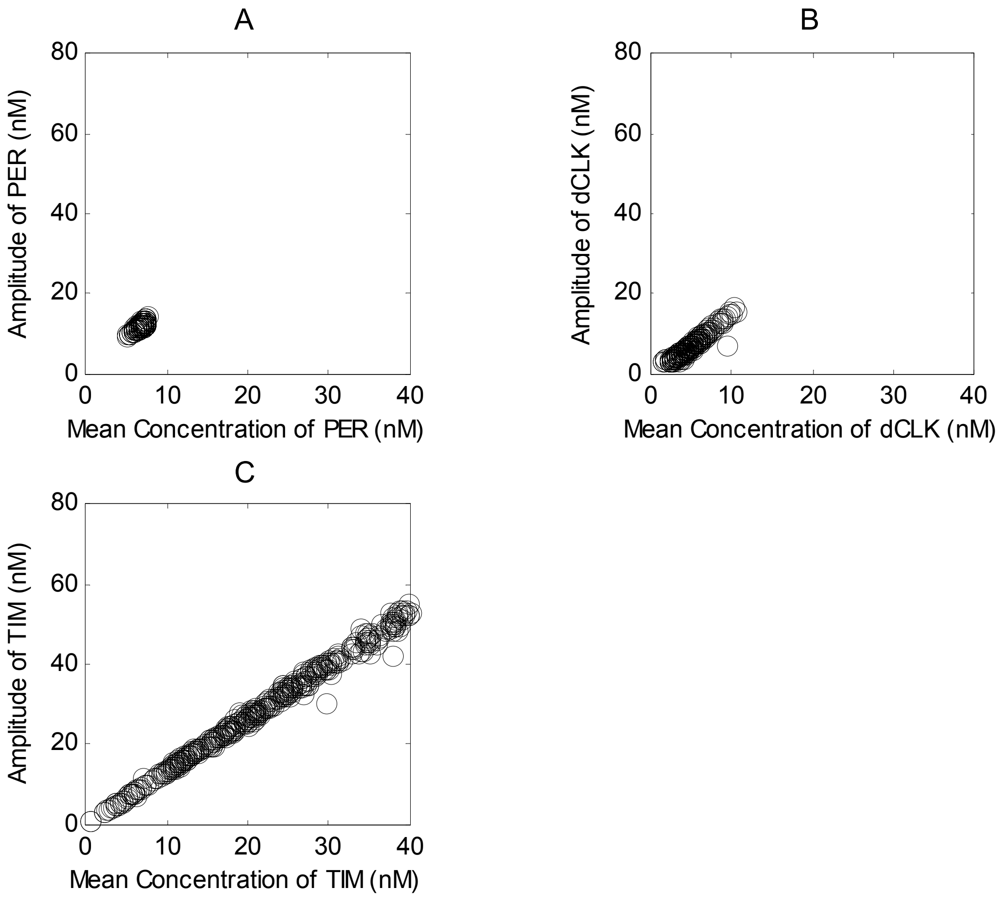

Figure S4 Characterization of oscillatory features for PER, dCLK, and TIM
We investigate the cycle features of PER, dCLK and TIM when the circadian model is optimized with regard to the PER oscillator. The amplitudes and the mean concentrations of PER (A), dCLK (B), and TIM (C) are plotted.
