# Supplementary material for: Mathematical Identification of Critical Reactions in the Interlocked Feedback Model
Source: PLoS One. 2007 Oct 31;2(10):e1103. doi: 10.1371/journal.pone.0001103 (PMC2040204; doi:10.1371/journal.pone.0001103)
Supplement: FigureS5 — (0.14 MB PDF) [file pone.0001103.s008.pdf]

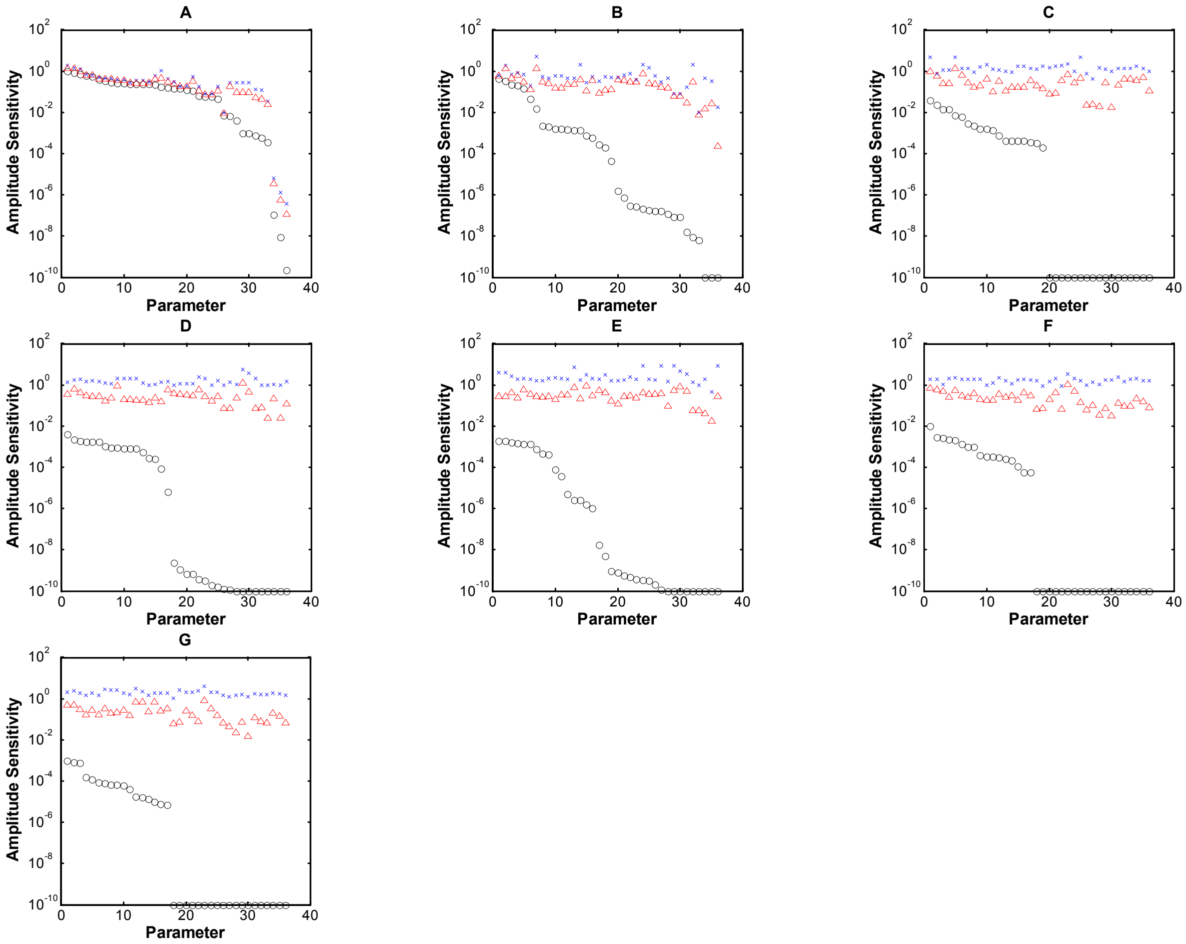

Figure S5 Effect of the number of search parameters on separation of potentially influential parameters

We investigated how the number of search parameters distinguishes the potentially influential parameters (**Equation 10**). For various numbers of search parameters, we generated 400 solutions optimized for a PER cycle and then simulated the absolute value of the amplitude sensitivity when each kinetic parameter was varied by 2-fold. The resulting distributions exclude the models whose oscillations are abolished by parameter perturbations ($|Sensitivity| > 10$). The amplitude sensitivities with a value of less than 10^{-10} are set to 10^{-10}. The kinetic parameters are sorted according to the minimum sensitivity (black circle) in the descending order. The mean value (red triangle) and the maximum sensitivity (blue cross) are also plotted. The label of the figures shows: (A) explores 3 parameters (*S[1], A[1], A[2]*),

(B) 6 parameters (*S[1], A[1], A[2], R[1], R[2], V[1]*),

(C) 9 parameters (*S[1], A[1], A[2], R[1], R[2], V[1], V[2], D[1], P[1]*),

(D) 12 parameters (*S[1], A[1], A[2], R[1], R[2], V[1], V[2], D[1], P[1], T[1], K[1], K[2]*),

(E) 16 parameters (*S[1], A[1], A[2], R[1], R[2], V[1], V[2], D[1], P[1], T[1], K[1], K[2],S[4],D[3],D[5],D[7]*),

(F) 25 parameters (*S[1], A[1], A[2], R[1], R[2], V[1], V[2], D[1], P[1], T[1], K[1], K[2],S[4],D[3],D[5],D[7] S[3], A[3], R[3], V[3], V[4], D[8], D[11], T[3], K[4]*),

(G) the entire 36 parameters.

The values of *T[2], T[4], K[3],* and *K[5]* are set to zero.
