# Supplementary material for: Mathematical Identification of Critical Reactions in the Interlocked Feedback Model
Source: PLoS One. 2007 Oct 31;2(10):e1103. doi: 10.1371/journal.pone.0001103 (PMC2040204; doi:10.1371/journal.pone.0001103)
Supplement: FigureS6 — (0.11 MB PDF) [file pone.0001103.s009.pdf]

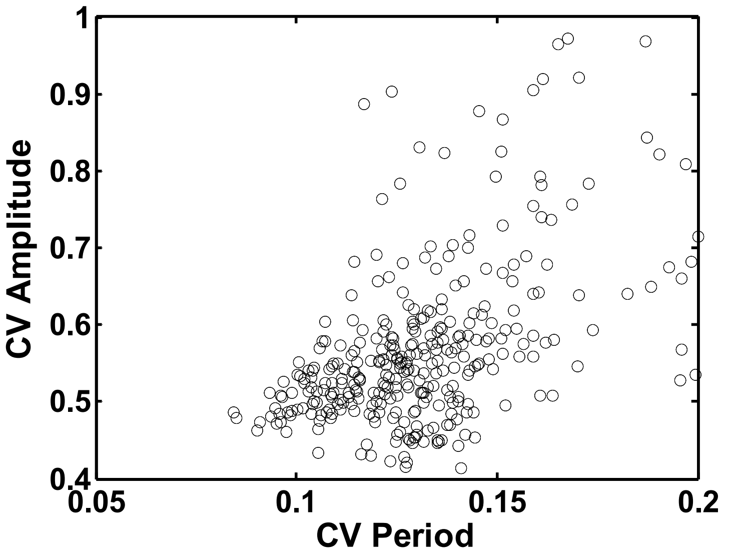

Figure S6 Relationship between the CVs of period and amplitude
The CVs for the period distribution generated by the random simulations are plotted with respect to the CVs for the amplitude distributions.

Significant linear correlation was not observed between the CVs of period and amplitude. The mechanism that provides a robust property to the period is suggested to be different from that to the amplitude.
