# Supplementary material for: Mathematical Identification of Critical Reactions in the Interlocked Feedback Model
Source: PLoS One. 2007 Oct 31;2(10):e1103. doi: 10.1371/journal.pone.0001103 (PMC2040204; doi:10.1371/journal.pone.0001103)
Supplement: FigureS7 — (0.10 MB PDF) [file pone.0001103.s010.pdf]

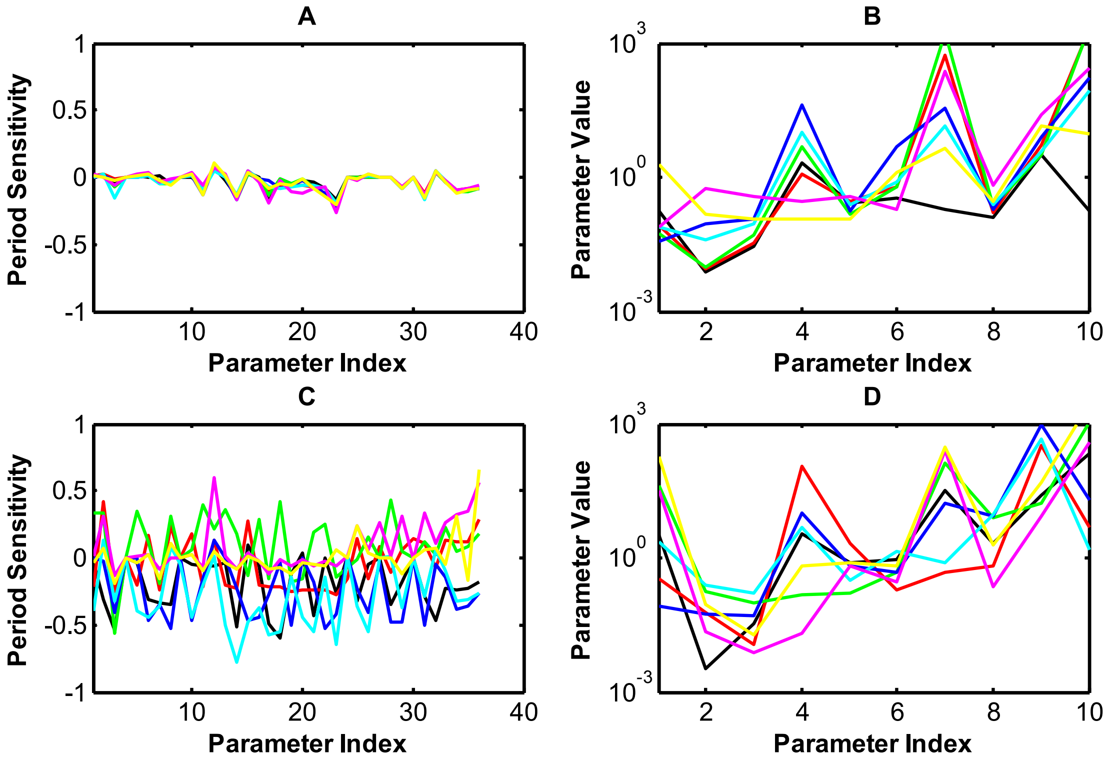

Figure S7 Distributions of period sensitivities and their associated kinetic parameter values

In the upper panels, the period sensitivity (A) and parameter value (B) distributions of the models showing seven smaller values of the CV period are plotted. A parameter index of one indicates *S[1]*, 2: *A[1]= A[2]*, 3: *R[1]=R[2]*, 4: *V[1]*, 5: *V[2]*, 6: *D[1]*, 7: *P[1]*, 8: *T[1]*, 9: *K[2]*, 10: *K[1]*.

In the lower panels, the amplitude sensitivity (C) and parameter value (D) distributions of the models showing seven larger values of the CV period are plotted.
