# Supplementary material for: Mathematical Identification of Critical Reactions in the Interlocked Feedback Model
Source: PLoS One. 2007 Oct 31;2(10):e1103. doi: 10.1371/journal.pone.0001103 (PMC2040204; doi:10.1371/journal.pone.0001103)
Supplement: FigureS8 — (0.10 MB PDF) [file pone.0001103.s011.pdf]

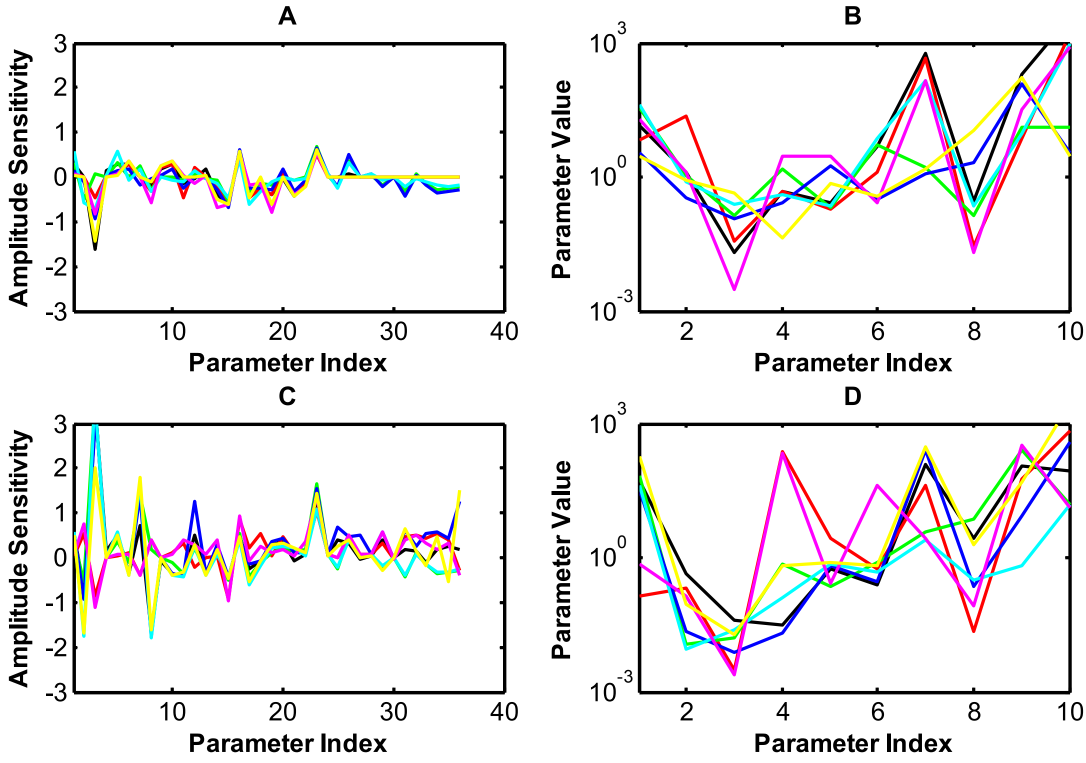

Figure S8 Distributions of amplitude sensitivities and their associated kinetic parameter values

In the upper panels, the amplitude sensitivity (A) and parameter value (B) distributions of the models showing seven smaller values of the CV amplitude are plotted. A parameter index of one indicates *S[1]*, 2: *A[1]= A[2]*, 3: *R[1]=R[2]*, 4: *V[1]*, 5: *V[2]*, 6: *D[1]*, 7: *P[1]*, 8: *T[1]*, 9: *K[2]*, 10: *K[1]*.

In the lower panels, the amplitude sensitivity (C) and parameter value (D) distributions of the models showing seven larger values of the CV amplitude are plotted.
