# Supplementary material for: Mathematical Identification of Critical Reactions in the Interlocked Feedback Model
Source: PLoS One. 2007 Oct 31;2(10):e1103. doi: 10.1371/journal.pone.0001103 (PMC2040204; doi:10.1371/journal.pone.0001103)
Supplement: FigureS9 — (0.12 MB PDF) [file pone.0001103.s012.pdf]

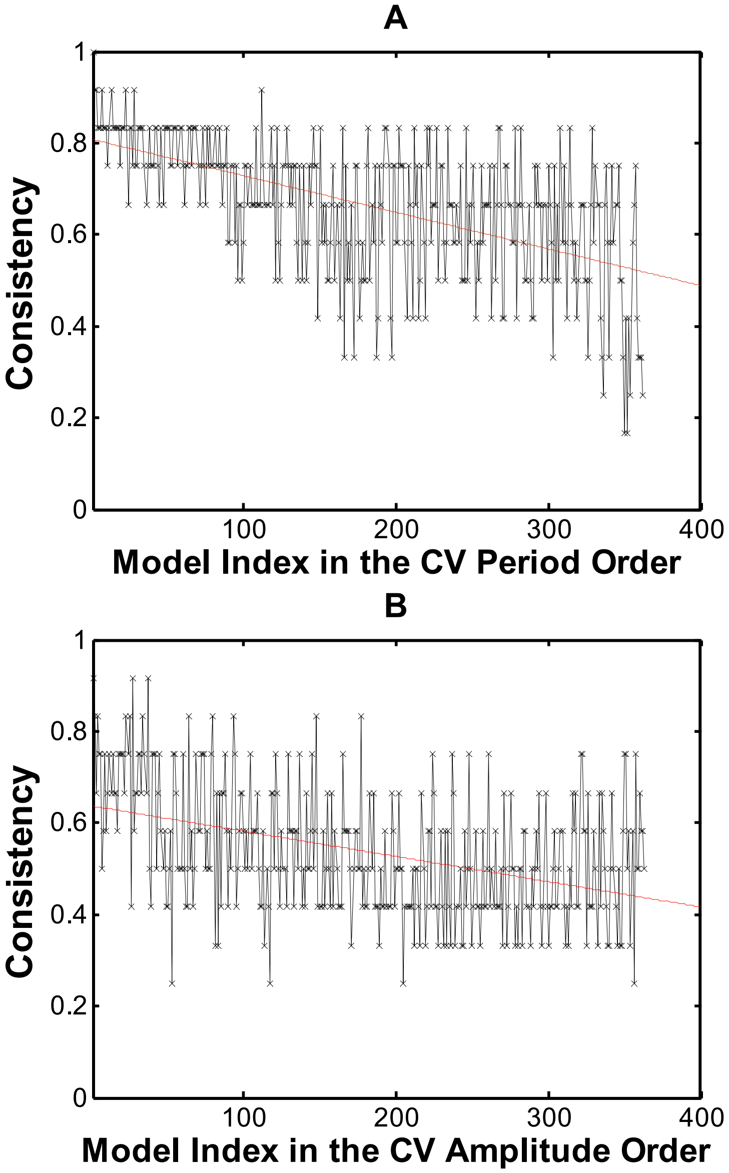

Figure S9 Consistency of the critical parameters responsible for determining period or amplitude
The twelve critical parameters for determining period and amplitude were selected from **Figure 9**, respectively. To investigate whether these twelve critical parameters feature robust oscillation, the consistency was defined. For each model, 12 kinetic parameters showing a higher absolute sensitivity were selected and the number of the selected kinetic parameters corresponding to the selected critical parameters was counted. The consistency is provided as the ratio of the number of the corresponding number to 12 and is plotted with respect to the models sorted in the ascending order of the CV values.
(A) Consistency of the twelve critical parameters responsible for period,
(B) Consistency of the twelve critical parameters responsible for amplitude.

With a decrease in the CV value, the consistency increases, indicating that the selected critical parameters feature highly robust models with a small CV value. The selected parameters are found significantly critical for determining period or amplitude in the highly robust models.
